# Confidentiality agreements: a challenge in market regulation

**DOI:** 10.1186/s12939-019-0916-3

**Published:** 2019-06-03

**Authors:** Roberto F. Iunes, Manuela Villar Uribe, Janet Bonilla Torres, Marina Morgado Garcia, Carolina Zampirolli Dias, Juliana Alvares-Teodoro, Francisco de Assis Acurcio, Augusto Afonso Guerra-Junior

**Affiliations:** 1 The World Bank, 1818 H Street, Washington, D.C. 20433 USA; 2 Department of Social Pharmacy, Graduate Program of Medicines and Pharmaceutical Services, Federal University of Minas Gerais, Av. Presidente Antônio Carlos, 6627, Pampulha, Belo Horizonte, Minas Gerais 31270-901 Brazil; 3 Department of Social Pharmacy, Public Health, Federal University of Minas Gerais, Av. Presidente Antônio Carlos, 6627, Pampulha, Belo Horizonte, Minas Gerais 31270-901 Brazil; 4 Department of Social Pharmacy, Federal University of Minas Gerais, Av. Presidente Antônio Carlos, 6627, Pampulha, Belo Horizonte, Minas Gerais 31270-901 Brazil

**Keywords:** Equity, Confidentiality agreements, Market regulation

## Abstract

**Background:**

Sustainability and the ability to maintain the right to health, with the guarantee of access to quality medicines and health services, have been a great challenge for countries with universal health systems. The great technological advances bring with it an expressive increase in the expenditures of the health systems, especially those directed towards the acquisition of high-cost drugs, which are still under patent protection, have a high cost and, in some cases, present uncertainties about their effectiveness and safety. As a way of maintaining the proper functioning of the systems and guaranteeing access to these medicines, some countries started to negotiate discounts with manufacturing companies. Pricing agreements have been adopted by developed countries with the objective of reducing their spending on high-cost medicines and, although they represent an opportunity for better negotiation with the industries, they violate the principle of transparency that regulates the world market. However, the existence of confidentiality agreements has meant that the declared prices are not the actual prices, unfairly harming the countries that use these price lists as beacons in their systems.

**Methods:**

Representatives of health, judicial, legislative, patient organizations and academics from eight countries in Latin America and South Korea participated in a meeting in September 2017 in Chile to discuss price confidentiality agreements and the impact on public health policies. During the meeting, participants were presented with a hypothetical case to subsidize the discussion on the topic. Divided into groups, participants should propose recommendations for the problem by pointing out the pros and cons if each proposed recommendation was adopted. The groups were then confronted by a simulated jury and finally issued a single and final recommendation for the problem.

**Results:**

The topic was widely discussed and recommendations were raised by the participants. Among them, it is worth noting the elaboration of norms that regulate the negotiations of prices between the countries bringing transparency and harmony in the adopted conducts. In addition, the possible consequences and potential impacts of confidentiality on drug prices and inputs, such as information asymmetry and inequity of access between countries, were pointed out.

**Conclusion:**

Despite there are efforts to make price negotiations more transparent, there is still no well-established standardization that promotes a well-functioning market. Confidentiality agreements hamper the fairness of access to essential health products.

## Background

The pharmaceutical industry is one of the most innovative in the market [8]. Several diseases have gained effective treatments, resulting in reduced mortality and morbidity of the population and increased life expectancy and quality of life [9]. These new drugs, although necessary, are often priced high and do not guarantee their effectiveness and efficacy. These factors, associated with the limited budget of universal health systems, represent one of the greatest challenges for managers in promoting fair and comprehensive health care [16, 5]. Therefore, many countries began to seek strategies to manage the entry of these drugs in the market, trying to minimize the impact on their budgets of expensive drugs without guarantee of expected results [3].

Among the used strategies, the agreements on the entry of medicines into the market stand out. These agreements are signed between a manufacturer and a public manager, in order to allow access to a specific medicine or health technology in specific conditions [2] and may involve simple discount on the unit price of a drug, discounts based on the use or performance, or both [13]. The most used types of agreements in European countries now include financial and performance agreements [1].

The most widely used financial arrangement is price reduction, which usually takes place through individual negotiations between public payers and pharmaceutical industries. Other types include refunds of securities by pharmaceutical companies according to the volume of sales; support in kind, in which you buy two and win one; grouping, a sales strategy that unites the products to sell them as a single combined unit; among others [16].

The performance agreement is a management pact when there is uncertainty about the efficacy and clinical cost effectiveness of the drug in question, for example, the case of the drug Lucentis, from Novartis, which was approved for marketing although the number of required injections is not known in the “real life”. The company took advantage of this approval to develop this new evidence about the drug, assuming that if not achieved the expected results, it would need to lower the purchase price [10].

In general, price contracts are presented, at first glance, as a profit opportunity for both buyers and the industry. However, when they are confidential, they represent an obstacle to transparency in the price negotiations for medicines, since the secret will be an ally for the manufacturer’s pricing [13, 16]. This situation is due to the fact that most countries make price comparisons with reference to official list prices. When there is confidentiality of the values practiced in the contracts, a situation will occur when prices are only an indication and do not really reflect the values practiced [7, 16].

Confidential pricing agreements are currently widespread in the world. Managers from countries in Europe, North America, Australia and Asia made these commitments to reduce the impact on the budget and to improve the use of new technologies. Although it represents an economy, this kind of activity may be indirectly violating drug list prices, as well as generating a scenario in which managers are forced to participate if they wish to obtain the best possible prices for the populations they serve [13]. Sustainability and the ability to maintain the right to health, with the guarantee of access to quality medicines and health services, have been a great challenge to managers. In this way, the discussion about confidential pricing agreements is important to contribute to a better understanding of the challenges and implications in the field of health and law on how to adopt practices for transparency in the negotiation and price agreements for medicines and health technologies by countries. This paper aims to describe the discussions held at the Sixth Meeting of the *SaludDerecho Initiative* of the World Bank and the Chilean Ministry of Health, in September 2017, in Santiago, Chile, to help countries to reach possible solutions to this problem.

## Methods

During the Sixth Meeting of the *SaludDerecho Initiative* of the World Bank and the ministry of health of Chile, held in september, 2017, in Santiago, Chile, 51 representatives of health, judicial, legislative, patient organizations, and academics from eight Latin American and South American countries were invited to debate about some issues that have impact on public health policies, such as confidential price agreements of health technologies. A hypothetical case was proposed about “Ethics and transparency in access to medicines”. This theme is one of the main challenges for the regulation of the global pharmaceutical market.

For the discussions, an adaptation of the technique known as the “Delphi Method” was used, as a systematized method of judging information, in which a consensus of experts is sought for decision making through articulated validations. This can be used to get any kind of consensus among people and involves identifying a problem and presenting the results [6]. The method takes place with a systematic communication structure composed by experts in some specific field and controlled by a group of researchers. In this, the experts receive feedbacks about the opinions raised, revising their opinion, in order to answer the points raised by another participant. At the end, the aim is to reach a consensus about the problem in question [14].

In this way, a study case addressed “*The development of a new drug – SE-MAB™ for breast cancer - and its efficacy and safety*” was elaborated to encourage the discussion about confidentiality agreements. In this, the Ministry of Health of many countries had received a confidential proposal for the incorporation of SE-MAB™ for breast cancer. Chile had a price 70% lower than the defined market price, Uruguay 50% lower and Costa Rica 40% lower. In contrast, all of then should keep this prices proposals confidential, adopt a centralized purchase scheme for the country, disclose to countries that the drug is being used in your country and keep the registry of patients who are using this for post-marketing publications.

The 51 participants were divided into working groups with the purpose to discuss the theme, answering the three questions proposed:

1. What are the consequences of the confidentiality of prices of health technology purchases for the countries?
2. What can courts and health ministries do to improve trade agreements and increase transparency in price negotiations with the pharmaceutical and supplies’ industries?
3. What are the pros and cons for the countries to adopt confidentiality agreements in the acquisition of health technologies?

After the discussion of these questions within groups, each of them should elaborate recommendations for this problem. Then, discuss in a plenary meeting the pros and cons each group pointed out in a simulated jury. After the discussions of pros and cons between the groups, they had to discuss the impact about adopting the recommendations they proposed.

**Fig. 1:**
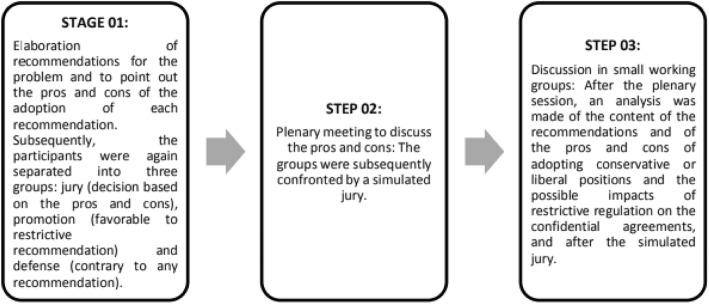
Stages of proposed activities

All the stages of the method adopted are summarized in the Fig. 1.

After the meeting, the collected data was analyzed qualitatively, adopting procedures of the content analysis. Initially, the information was coded, whereby raw data are systematically transformed and grouped into units that allow the description of relevant characteristics of their content. Thus, the main ideas cited were extracted or inferred from the textual data, categorizing their content. Finally, the main results and conclusions of the groups were described and discussed [4].

## Results

Following the stages of the proposed activities, the participants answered to each question. The first question, asking about the consequences of the confidentiality of prices, the groups highlighted asymmetry of information, that may favor the manufacturer; iniquity of access and masking of monopolistic practices. For the second, about what courts and health ministries can do to improve trade agreements and increase transparency in price negotiations, they told about making public the market prices, to make purchase agreements between countries; pricing, coefficients and rates fixed, payment of fines in case of noncompliance, and establishment of specialized courts in health.

At the end, seven recommendations were pointed out by the participants. The main recommendations took into account aspects related to the transparency of health technology prices in relation to the negotiation of prices with producers of medicines and supplies. In addition, the importance of efforts by the executive, judiciary and also the Ministry of Health to enhance trade agreements was stressed. Furthermore, according to the methodology used in the activity, the pros and cons regarding each proposed recommendation were raised. The results are detailed in the Table 1.

**Table 1:** Description of the recommendations with pros and cons

Number	Description of the recommendation	Pros	Cons
01	Transparency in the prices of sanitary technologies	Larger negotiating margins for countries	This would lead to the same price in the world that would produce iniquity
		Promote integration between countries for the purchase of medicines and supplies	Trading limiting prices for small countries or countries with less bargaining power
		Reduces corruption in drug purchases	-
		It favors competition (when there are alternative products)	-
		The prices would bear more relation with the costs	-
02	Efforts of the Ministry of Health and Executive to improve trade agreements and transparency in negotiating prices with the drugs’ and supplies’ industry.	Concentrate purchasing and trading with industry across the country	Discourages the industry to sell in that country
		Define purchase prices for universal coverage	Impossibility for the industry to offer low prices to low-income countries
		The price must adjust (principle of equity) to the economic possibilities of the countries	-
		Do not purchase a drug without health technology assessment, budget impact and cost effectiveness	-
		Ministries of health should participate in the negotiation of free trade agreements	-
03	Judiciary efforts to improve trade agreements and transparency in the negotiation of prices with the drugs’ and suplies’ industry	To interpret trade agreements in the light of the right to health of people and access to health information (including prices for the purchase of medicines)	This could cause negotiation problems for the Executive or the health authorities
		Prevent the access, through justice, to the market of products or supplies that have not undergone the health evaluation or have incorporated health services.	
04	Inclusion of the obligations assumed by the State as a counterpart of the discount on the price of the drug	The immediate discount on the price	Eliminate competition
		Improvement of medication due to use of patient outcomes	Prevents the state from making an informed decision
05	Approve rules regulating this type of negotiations, requiring transparency and expressly stating that neither the price nor the other terms of the agreement can be confidential, except those protected by trade or industrial secrecy	-	Favors corruption because it is not transparent
06	Propose methodologies and train staff to address the price formation debate	-	Conditions public health policies
		-	The use of the drug is improved without the patient having authorized the use of the results obtained by providing that medicine
07	Confidentiality agreements on the acquisition of sanitary technologies	The supposed fall in prices	Breaks the parameters of a joint trading policy
		Access to the latest generation of drugs, which can not be accessed in any other way or by any other type of negotiation.	Lack of transparency
		-	Blocks social control
		-	It hinders and damages competition: there are no other suppliers
		-	Another industry can’t present a better offer
		-	There is no guarantee that the best price is being charged
		-	Cross subsidies: the highest paid country can pay a part of the country that pays less
		-	The industry ends up putting the rules of the game: the country enters the industry game

After the discussions, the experts proposed the development of clear and transparent rules to promote a smooth functioning of the market as a final recommendation to solve the problem of confidentiality agreements and their consequences between countries.

## Discussion

Confidential pricing agreements is challenging to health and law authorities. In this way, pointing out recommendations may help in solve these issues, being a potential guidance to countries to pass through these problems. So, the development of rules in this context is important, since those are medicines and health technologies, having impact in public health.

In this way, pharmaceutical market regulation occurs in response to market failures when there are discrepancies in relation to the ideal of a competitive market [11]. Usually, it is performed as a state intervention in the economic sphere. Although there are already some rules in the market that can bring transparency to the negotiations, there is still secrecy and confidentiality of the same in most countries. Examples are Belgium and France, where agreements of oncology drugs used to be strictly confidential [15].

Likewise, confidentiality can be an ally for pricing by the manufacturer, resulting in different prices, even higher than those charged in other countries, resulting in high profits for producers. In addition to confidentiality, industry also relies on the fact that there is no production of some drugs in its subsidiaries in underdeveloped countries, which only import such drugs from abroad, presenting this to justify a difference in prices between countries [12].

It is known that the consequences of this confidentiality can also lead to the emergence of monopolies and asymmetry of information, that is, separation of decisions on prescription, consumption and financing of medicines. Thus, the drug market becomes imperfect, leading to unequal competition and price changes, and there is no doubt about the need of effective regulation in the marketing of medicines by countries [12].

It also worth noting that the difficulty of access, together with the question of the medicines being a health product could not, therefore, be seen as a commercial good. This entails the need for state intervention in the guarantee of access, avoiding that there are financial abuses in the commercialization of these products. In cases this may occur, an important strategy would be to set and adjust prices by setting margins for both sides so that everyone benefits theirself and have their interests met. In addition, mechanisms facilitating procurement notifications that seek to improve communication among government entities are of fundamental importance in order to avoid asymmetry of information and inequality of access between countries.

It is important to highlight that the results found in this paper may present limitations since there were representatives from only 8 countries, including Latin America, South America and Asia, which may not reflect the opinion of the countries and other representatives.

## Conclusion

Despite there are efforts to make price negotiations more transparent, there is still no well-established standardization that promotes a well-functioning market. Confidentiality agreements hamper the fairness of access to essential health products. From the recommendations, it is necessary to elaborate norms that regulate the negotiations of prices between the countries, bringing greater transparency and harmony in the adopted conducts.
